# Case report: Personalizing the use of trazodone in real-world patients: a study of three cases of depression with comorbidities

**DOI:** 10.3389/fpsyt.2024.1362221

**Published:** 2024-08-29

**Authors:** Gianluca Rosso, Beatrice Benatti, Mauro Pettorruso, Gaia Sampogna, Carmine Tomasetti

**Affiliations:** ^1^ Department of Neuroscience, University of Torino, Turin, Italy; ^2^ Department of Biomedical and Clinical Sciences Luigi Sacco, Luigi Sacco Hospital, University of Milan, Milan, Italy; ^3^ CRC Aldo Ravelli, University of Milan, Milan, Italy; ^4^ Department of Neuroscience, Imaging and Clinical Sciences, “G. d’ Annunzio” University, Chieti, Italy; ^5^ Department of Psychiatry, University of Campania “L. Vanvitelli’, Naples, Italy; ^6^ Department of Mental Health of Teramo, ASL Teramo, Alzheimer Centre of Giulianova, Teramo, Italy

**Keywords:** SARI, SSRI, comorbidity, Parkinson’s disease, sexual dysfunctions, cognitive decline

## Abstract

Depressive disorders are leading contributors to the global mental health-related burden, and they represent a challenge for real-world clinicians, due to the low rates of remission despite the high availability of treatments. Often, depression shows in the context of multiple chronic comorbidities, thus requiring precise and accurate management of pharmacological treatments to avoid interactions and side effects. These criticalities call for the need for new strategies of treatment, which may include new insights into the pharmacological properties of currently available antidepressant drugs, to enhance their efficacy in the different contexts in which depression may arise. Trazodone is the prototype serotonin antagonist/reuptake inhibitor antidepressant (SARI). Due to the malleability granted by its multiple formulations, trazodone is frequently used to treat depression, both as an add-on to other antidepressants and as a monotherapy, with satisfying results. Moreover, its tolerability makes it one of the most prescribed antidepressants in patients with poly-treated comorbid medical illnesses, especially in the elderly. Herein, a case series is presented regarding the use of trazodone in patients with complex comorbid diagnoses or distressing side effects. Each of the three cases has been discussed in three specific Round Tables, involving expert clinicians in the fields of Psychiatry, Neurology, General Practice, and Geriatrics using the Nominal Group Technique. The ideas collected have been used to integrate the cases and the discussion with the intent of facilitating accessibility to the widest audience of physicians and clinical workers in different clinical practice contexts. The final aim of this paper is to promote an increasingly personalized use of trazodone in real-world patients with depression.

## Introduction

Depressive disorders represent the leading contributors to mental health-related global burden, with an estimated 3.8% of adults affected worldwide, and a suicide rate of 700.000 people per year (1). Globally, depressive disorders have been calculated to lead to 5 million years lived with disability (YLD), thus being ranked as the major cause of non-fatal health loss (7.5% of all YLD) (2). In Italy, patients diagnosed with depressive disorders represent the most prevalent population utilizing mental health services (34.6/10.000 people), with women almost doubly more affected than men (43.2 vs 25.4/10.000 people), thus requiring high costs, in terms of personnel and treatments employed (3). Depressive symptoms have been reported in one out of 10 primary care patients (4), and they often arise in the context of many other comorbid disorders, such as endocrine dysfunctions (e.g., hypothyroidism, Cushing syndrome), cardiovascular disorders, stroke, Parkinson’s Disease (PD), and major neurocognitive disorders (e.g., Alzheimer’s Disease), thus complicating their progress and treatment, as well as increasing their burden and worsening outcomes (5, 6). Thus, depression represents a challenge for real-world clinicians, due to the low rates of remission despite the increasing number of antidepressant strategies currently available (7, 8). Consolidated evidence, indeed, supports the view of envisioning depression as a mind-body disorder, trying as much as possible to tailor the antidepressant treatments to the individual features of the patient, such as their physical characteristics, psychological fragilities, comorbidities, and pharmacological treatments, and combining pharmacological and psychotherapeutic treatments to maximize their effectiveness (9, 10). Regarding pharmacological treatments, the aforementioned criticalities call for the optimization of the efficacy of current antidepressants. Although SSRIs (selective serotonin reuptake inhibitors) are the first-line treatment in depressive disorders, they often fail to achieve acceptable rates of remission (11). Thus, multiple strategies have been proposed to maximize antidepressants’ efficacy, such as add-ons with other mood stabilizers, antipsychotics, GABA modulators, and combined psychotherapies (12).

A valuable approach may indeed be the optimization of current antidepressants, by means of taking advantage of new insights into their pharmacological properties, as well as in the possible use of multiple formulations, in order to enhance their efficacy in different contexts. According to this approach, here we present three case reports in which trazodone, an “old” antidepressant with several peculiar characteristics, has been used to manage depression in complex patients to highlight the real-world implications for clinical practice.

Trazodone is a triazolopyridine derivative, developed in Italy by Angelini Research Laboratories in the 1960s as a second-generation antidepressant, and it was the prototype serotonin antagonist/reuptake inhibitor antidepressant (SARI). Trazodone has the pharmacological property of blocking 5HT2a/5HT2c serotonin receptors and inhibiting serotonin transporter (SERT) activity (13). When administered in low doses, trazodone potently blocks 5HT2a receptors and preferentially antagonizes histamine 1 and alpha 1 adrenergic receptors, thus exerting powerful anxiolytic and sedative/hypnotic effects. At medium/high dosages, trazodone exerts its complete action by blocking the serotonin transporter (SERT) and 5HT2a/2c receptors (13).

Trazodone is also extremely versatile when used as an add-on to other antidepressants, both SSRIs and SNRIs (serotonin/noradrenaline reuptake inhibitors), providing complementary effects. Indeed, some studies reported an increase in antidepressant effects when adding trazodone to an SSRI as compared to the latter alone, most likely due to trazodone’s anxiolytic/sedative/hypnotic properties (14). Lastly, trazodone has demonstrated progressive antidepressant effects on peculiar symptom domains, such as mood and energy loss. This action is exerted by incrementally recruiting 5HT1a/2c receptors and SERT through several mechanisms of action: 1) the 5HT2a blockade may potentiate the inhibitory action of 5HT1a receptors, inducing anxiolytic effects; 2) the activation of 5HT1a receptors may promote neurotrophic factors’ gene expression, relieving depressive symptoms; 3) 5HT1a receptors may inhibit the abnormal firing of some cortical glutamate pyramidal neurons, which are considered responsible for cognitive symptoms in depression; 4) 5HT12a and 5HT2c blockade promotes the disinhibition of noradrenaline and dopamine release in the cortex, thus contrasting prefrontal symptoms of depression (13).

Currently, trazodone is available in three different formulations: immediate release (IR), prolonged release (PR), and once-a-day extended release (OAD). Trazodone IR has a rapid plasma peak (1 hour) and a short half-life (6.6 hours); trazodone PR has a slower plasma peak (4 hours) and a longer half-life (12 hours), and trazodone OAD shows a plateau plasma level for the entire day, with longer antidepressant concentration as compared to the other formulations (14).

Due to its longevity as an antidepressant, considerable evidence exists of the efficacy of trazodone in treating depression. Indeed, several studies described an antidepressant efficacy equivalent to that of SSRIs when compared to placebo, albeit the older formulations demonstrated higher rates of side effects and drop-outs (15). The introduction of the recent OAD formulation granted trazodone a considerably higher antidepressant efficacy as compared to placebo, with side effects comparable to (when not lower than) those observed for other antidepressants (16, 17).

Since its first distribution, trazodone has demonstrated a unique profile of tolerability, and it rapidly stood out as one of the most prescribed antidepressants in patients with depression with poly-treated comorbid medical illnesses, especially in elderly people (18). Lower rates of sexual-related side effects compared to SSRIs have also been demonstrated (19). Moreover, increasing evidence suggests a good control of behavioral disorders, as well as pro-cognitive effects, in depressed patients with comorbid neurodegenerative disorders (20). Last, in real-world settings, trazodone has been found to exert a faster-onset antidepressant effect compared to other antidepressants, with sustained relief after 6 weeks, both in drug-naïve patients and in those unresponsive to previous treatments (21–23).

Taken together, all these properties make trazodone a valuable choice in the treatment of depression in various contexts. Its multimodality of action and the multiple available formulations help treatment optimization to meet specific unsatisfied needs.

Hereafter, we will focus on three cases of patients diagnosed with depression, presenting with diverse comorbidities, which were assessed in Italian clinics, and in which trazodone was chosen as antidepressant treatment based on its peculiar multimodal characteristics.

## Methods

The three patients described have been assessed and treated respectively at: 1) The Department of Psychiatry, University of Campania “L. Vanvitelli”, Naples, Italy; 2) The Department of Biomedical and Clinical Sciences Luigi Sacco, Luigi Sacco Hospital, University of Milan, Milan, Italy; 3) Department of Neuroscience, Imaging and Clinical Sciences, “G. d’Annunzio” University, Chieti, Italy. The patient data have been appropriately anonymized, and all the patients provided written consent to the publication of their cases. When standardized methods were used for the clinical and instrumental assessment of patients, they are detailed in the respective descriptions.

Each of the three cases was discussed in three specific Round Tables, involving expert clinicians in the fields of Psychiatry, Neurology, General Practice, and Geriatrics using the Nominal Group Technique (NGT) (24). Three facilitators (one from each clinic in which the cases were assessed), coordinated by a master facilitator, prepared the presentation of the cases and the NGT questions and summoned the experts for the Round Tables. Eight expert physicians per Round Table were chosen based on their expertise in the aforementioned fields, and selected according to the macro-region of Italy in which they usually work (i.e., North, Center, and South). Thus, in each Round Table, respectively denominated North, Center, and South, the experts were invited by the facilitators to discuss the diagnostic and therapeutic paradigms used to assess and treat patients, with specific questions (NGT questions and their answers are reported in **Supplementary Table 1**). Then, they debated the clinical insights of the treatment choices. The ideas that emerged during the Round Tables were discussed according to the expertise of the physicians, and the answers to the NGT questions were ranked using a computerized tool to calculate the percentage of preferences by the experts in each table. After ranking the ideas emerging from the discussion, as well as the answers, these were collected in specific reports and used to integrate a case series to facilitate accessibility and grant an extended usability of the information to the widest possible audience of physicians and clinical workers in different “real-world” clinical practice contexts.

## Presentation of cases

### Case report A: An elderly patient with major depressive disorder and cognitive decline

Patient A was a 77-year-old woman. She had been a widow for two years; thus, she came to the visit with her daughter. Since her husband’s death, she had lived alone, and her daughter had lived in another city. Her daughter insisted on scheduling the visit because she noticed a behavioral change in her mother during the last 6 months: frequent crying breakdowns, loss of interest in daily activities, and reduced self-care. The patient reported difficulties in falling asleep, as well as in maintaining sleep and disabling levels of anxiety. The patient’s history included a diagnosis of hypertension (with poor compliance to pharmacological therapy), knee osteoarthritis, and hypercholesterolemia. Concerning her psychiatric history, the patient reported a previous depressive episode when she was 25 years old, with anxiety symptoms, in conjunction with her second daughter’s birth. At that time, she felt depressed, experienced a loss of interest in daily activities, and a sense of guilt because of her incapacity to manage the newborn, as well as fear of being unable to recover. Supported by her husband, she went to a specialist, who prescribed an unspecified therapy, with beneficial effects after 8-10 months. The patient brought no previous medical records, but she referred no further episodes until the current one. She described herself as a “woman who did nothing good” in her life. She alluded to herself as an “awful mother”, who forgot to pick up her 5-year-old son from school, for which she was rebuked by the teacher, and was never able to forgive herself. Moreover, she described herself as a “frail woman”, completely dependent on her husband; she did nothing good in her life and she was still mourning his death. The patient admitted using alcohol to manage her difficult personal conditions. She affirmed that at least twice a day for the last five years she has drunk almost four glasses a day of an Italian herbal liqueur (28% Alcohol by Volume – ABV). Her daughter confirmed such a maladaptive behavior of her mother and many times she has tried to force her to stop drinking, but all these attempts were unsuccessful. The interview was frequently interrupted by the patient’s crying breakdowns.

Patient A was diagnosed with a major depressive episode, moderate, with anxious distress (25). For a complete characterization of the clinical picture, an encephalic MRI was performed, together with a SPECT. Both showed a clinical picture of an ongoing organic process of cognitive decline with temporal lobe hypometabolism.

The complex comorbidity of depression, anxiety, cognitive decline, hypertension, and hypercholesterolemia led the clinician to preferentially start treatment with a SARI antidepressant drug. In particular, the presence of previous depressive episodes and the alcohol abuse was particularly relevant to be taken into consideration when choosing the antidepressant treatment. To avoid cross-interactions with other treatments and to manage mood alterations, anxiety, and the tendency to use alcohol, trazodone was selected. Indeed, a recent trial has highlighted that trazodone is effective not only in reducing depressive symptoms, but also in improving anxiety symptoms, sleep alterations, and cravings (26). Thus, Patient A was prescribed trazodone IR 50 mg, two tablets a day (breakfast and dinner) for 10 days, and then three tablets a day (breakfast, lunch, and dinner). After 20 days of treatment, the patient reported a reduction in anxiety levels and improved sleep. She felt less scared of staying home alone but guilt and feelings of worthlessness persisted. Hence, clinicians decided to upgrade the therapy with trazodone PR 150 mg twice a day (breakfast and dinner). After 2 months, the patient reported a significant reduction in anxiety and internal anguish, with a residual sadness due to the distance from her daughter. Some attention deficits persisted, probably due to the organic cerebral decline observed on the MRI and SPECT.

### Case report B: A young patient with sexual side effects from antidepressant treatment

Patient B was a 37-year-old man. He came to the clinic reporting feelings of incurability, anxiety, lower limb paresthesia, insomnia, and distressing sexual problems. At the time of the visit, he had interrupted his job. Concerning his medical history, he reported normal psychophysical development and diet, regular bowel and diuresis, and neither tobacco nor alcohol use, although he reported occasional cannabis smoking in the past. He referred allergy to acetylsalicylic acid and paracetamol. He had two siblings: his 34-year-old sister was in good health, while his first brother died at 2 months old. His mother suffered from dyslipidemia and hypothyroidism, whereas his father died at 63 years old because of myocardial infarction. The patient described a family history of psychiatric disorders: his paternal grandfather was diagnosed with generalized anxiety disorder; his father suffered from an unspecified anxiety disorder, and he was described as particularly apprehensive with regards to his son after the death of his firstborn, and he had a negative and demanding attitude towards his own family; his sister suffered from anxiety, depression, and panic attacks, currently treated with paroxetine.

Patient B reported that he began suffering from social anxiety when he was 18 years old, that he was prescribed paroxetine 20 mg/day, and that he underwent cognitive behavioral psychotherapy (CBT), with positive effects. Nevertheless, he autonomously stopped both treatments at 22 years old. When the patient was 24 years old, his father died, and he again experienced anxious states with somatic correlates and a slight hypochondriac ideation. However, until the summer of 2022 (at 37 years old) he had sufficiently managed his anxiety and maintained good social and work functioning without feeling the need to ask for medical advice. Then, he developed high levels of anxiety with somatizations, followed by depressed mood, feelings of inadequacy, difficulties in starting the day and in managing work and social appointments, clinophilia, social withdrawal, emotional lability with crying outbursts, and feelings of guilt and incurability associated with ideas of death, without actual suicide intention or planning. Moreover, he had reduced appetite and sleep. Thus, he was treated with paroxetine 40mg/day and started CBT again. After an unspecified time, he reported no benefits from the therapy and experienced significant weight gain, thus he was switched from paroxetine to sertraline 100mg/day. This was the last therapy he reported when first assessed in our evaluation.

In order to accurately assess the patient, he was administered with the following psychometric scales: the Hamilton Depression Rating Scale [HDRS, 17 items (27)], scoring 24 (indicating moderate/severe depression); the Hamilton Anxiety Rating Scale [HARS, 14 items (28)] with a score of 22 (moderate anxiety); Clinical Global Impression Scale [CGI-S (29)], with a score of 5 (markedly ill patient). Moreover, he was also administered with the International Index of Erectile Function (IIEF-5). IIEF-5 is a five-question self-administered questionnaire that assesses two domains: erectile function and sexual satisfaction. It was developed by the National Institute of Health’s Consensus Panel as an abbreviated version of the original IIEF 15 items questionnaire, in order to facilitate the assessment of the possible presence and severity of erectile dysfunction (30). It is composed of five self-administered questions with 1 to 5 Likert scale answers. A score >22 indicates no dysfunction, 17-21 slight dysfunction, 12-16 from slight to mild dysfunction, 8-11 mild dysfunction, and 5-7 severe dysfunction.

Patient B reported a score of 7 (severe difficulty in erectile function). Due to the important sexual dysfunctions and the persistence of depressive and anxious symptoms, the patient was offered to reduce sertraline to 50 mg/day (with the purpose of gradually stopping it) and to introduce trazodone OAD 150 mg in the late afternoon. A telephone interview was scheduled after 4 days. The patient reported an improvement in anxiety and sleep problems, but he was still depressed, unable to make decisions, and overwhelmed by work appointments. Thus, trazodone was increased to 225mg OAD. After 3 days, the patient reported stationary symptoms, thus trazodone was increased again to 300mg OAD, and sertraline was reduced to 25 mg/day and then suspended after 3 days. After 1 month, patient B reported a progressive improvement in his mood (HDRS=13) and a significant reduction in anxiety (HARS=10), as well as an amelioration of sexual dysfunction (IIEF-5 = 16), with global benefit to his symptoms (CGI=3) and to his work and social functioning.

### Case report C: A patient with Parkinson’s disease and major depressive disorder

Patient C was a 66-year-old man. He went to the clinic with his wife, reporting a progressive mood lowering in the last 12 months, associated with anxiety, clinophilia, poor self-care and personal hygiene, with apathy and anhedonia. He was greatly worried about his health and about his family’s economic conditions, which seemed unjustified to his wife. Additionally, he suffered from a disrupted sleep-wake pattern, with frequent night-time awakenings and excessive sleepiness during the day. His face was hypomimic, and his psychomotricity was greatly slowed down, although he was reported to have frequent restlessness episodes. Finally, he had postural instability, tremors (mainly at rest), and bradykinesia. Regarding his personal history, patient C was diagnosed with major depression at 25 years old, with different episodes, always successfully treated with SSRIs (escitalopram, sertraline) or SNRIs (venlafaxine) and low-dose antipsychotics (olanzapine), alternating with long periods of well-being.

He was married, had two siblings, and was described as a sociable, extroverted, and extremely active person. His wife reported the worsening of the disorder following his work retirement, with an increase in worries relating to the family’s economic conditions and a progressive reduction in social interactions. These depressive symptoms were accompanied by a significant psychomotor slowdown associated with postural instability and tremors at rest over the following months. He also presented bradykinesia. For these symptoms, the General Practitioner requested a neurological evaluation, which made a diagnosis of Parkinson’s Disease.

Thus, the patient was treated with a dopamine agonist (pramipexole) about 6 months before their visit, with notable improvement in motor symptoms and a reduction in apathy and anhedonia, whereas his depressed mood and alteration of the sleep-wake rhythm persisted, and anxiety psychomotor restlessness appeared to increase. The current depressive episode was treated with an SSRI drug (sertraline) and was subsequently replaced with an SNRI (duloxetine), in the absence of benefits and with the persistence of the symptoms mentioned.

The following diagnosis was formulated: major depressive disorder, recurrent episodes, severe; treatment-resistant depression; Parkinson’s Disease. He underwent a PET-DAT SCAN (positron emission tomography-dopamine transporter scan) exam, confirming the neuromotor disease for a reduced asymmetric uptake of the dopamine transporter at the level of the subcortical regions.

Considering the patient’s clinical picture (marked anxious component, psychomotor restlessness) and the poor clinical response obtained by using SSRI and SNRI drugs, it was decided to introduce trazodone 150 mg OAD (in the late afternoon) as an additional treatment. The choice of trazodone OAD was based on its controlled release over 24 hours, which makes it particularly suitable for patients with major depression, even severe, who can benefit from the possibility of starting the medication at a dose that is already potentially effective (150 mg), which can be rapidly increased to 300 mg (20). After a week, his anxiety and depressed mood appeared to be improving. He also seemed to have fewer concerns regarding his health and economic aspects. However, episodes of psychomotor restlessness persisted, mainly at night, and were associated with confusion and insomnia. Therefore, trazodone was increased to 300 mg OAD. Approximately 1 month after starting the treatment, patient C’s clinical picture appeared significantly improved: mood was slightly depressed, the amount of anxiety previously noted was significantly reduced, and there was noticeably less psychomotor tension. Finally, his wife reported a significant improvement in his sleep-wake rhythm, with the cessation of restlessness and confusion episodes during the night.

## Discussion

The case studies highlight the intricate nature of real-world healthcare challenges, reinforcing the notion that practical clinical application demands the incorporation of insights obtained from clinical trials. This complex clinical landscape is characterized by a spectrum of medical comorbidities and the imperative to mitigate and avert adverse effects that could hinder patient adherence to treatment regimens. Clinicians, therefore, require sophisticated and precise instruments designed to accurately target therapeutic goals and outcomes.

All three case reports can be considered representative of different clinical presentations of major depression and of the use of trazodone in clinical practice, based on its unique dosage-related mechanism of action and its good side effect profile and tolerability. Indeed, the three cases were chosen based on the “real-world” challenges of clinical management of depression.

As described in our first case report, depression has been globally reported in 35.1% of older adults (31), and often it emerges in the context of multiple comorbidities (e.g., cardiovascular diseases, vitamin deficiency, diabetes, etc.) which contribute to physical and cognitive impairment (32). Moreover, concurrent alcohol and drug use have been frequently reported in older adults, which may determine poorer outcomes and impaired responses to treatments (33). Finally, physicians treating depression in older patients often have to deal with complex poly-treatment schemes, which represent a further challenge in the choice of effective and safe antidepressant treatments (18). As in Patient A, trazodone is frequently prescribed in complex comorbid patients, above all elderly people, with the aim of managing mood and behavioral alterations. Recent studies have demonstrated that trazodone is efficacious in relieving depressive and anxious symptoms in elderly patients affected by dementia (20), primarily by restoring the sleep architecture (34), thus reducing the caregivers’ burden without impacting the cognitive function of patients (35). Moreover, trazodone has been associated with the prevention of cognitive decline in long-term users (36). Having a variety of formulations allows for flexible utilization of trazodone, as seen in the presented cases, by taking advantage of the peculiar pharmacokinetic/pharmacodynamic properties of each formulation. Currently, trazodone has been marketed in three different formulations: immediate release (IR), which has a rapid plasma peak (1h) and a short half-life (6.6h); prolonged release (PR), which shows a slower plasma peak (4h) and a longer half-life (12h); once-a-day (OAD) formulation, which has been demonstrated to maintain a plateau plasma level during the whole day, thus granting a more prolonged “antidepressant concentration” as compared to the other formulations (14). Although IR and PR/OAD formulations have been demonstrated to exert comparable antidepressant effects, with small efficacy measures favoring prolonged release formulations (37), significant differences may be noticed in the recruitment of specific receptors by the diverse formulations, due to their peculiar pharmacokinetics. Indeed, IR formulation, even when administered at low doses (30 mg), has been demonstrated to primarily recruit 5-HT1A, 5-HT1D, 5-HT2A, and 5-HT2B serotonergic receptors and α1A, α1B, and α1D adrenergic receptors, thus explaining the clinically observed efficacy of trazodone IR in treating insomnia and anxiety. Conversely, PR and OAD formulations progressively exert 5-HT2A and α1A antagonism and 5-HT1A partial agonism when administered at low doses, and then may block the SERT, H1 histaminergic, and 5-HT2C and 5-HT7 serotonergic receptors at high doses (150-300mg), thus gradually reaching the full antidepressant activity, with trazodone OAD maintaining these effects all through the day as compared to traditional trazodone PR (38). The different formulations’ pharmacokinetics may also explain the higher propensity of the IR formulation to induce drowsiness and dizziness, as compared to PR and OAD, although all formulations have demonstrated comparable safety and tolerability at both low and high dosages (39).

Therefore, as presented in Patient A’s case, starting with the IR formulation may help to control the main anxious symptoms and sleep disorders, and then switching to PR and OAD formulations may progressively achieve the desirable antidepressant effects without the disturbing side effects at higher dosages (39).

As reported in our second case, sexual dysfunctions have been regarded as one of the most disturbing side effects of SSRIs and SNRIs, as well as a potential cause of poor quality of life, nonadherence, and discontinuation, thus impacting patients’ potential recovery (40). Although well-known, iatrogenic sexual side effects continue to be underreported by patients and are often not sufficiently considered and emphasized by clinicians (41). Choosing an effective antidepressant strategy should consider the delicate balance between the efficacy for depressive symptoms and sexual tolerability, in order to facilitate adherence and therefore the complete recovery of treated patients, especially young patients (42).

Trazodone has been associated with lower rates of sexual side effects as compared to SSRIs/SNRIs (43), and low dosages (50-100mg) have been reported to partially relieve sexual impairments in patients taking SSRIs (44). Indeed, in some studies, trazodone has been reported as being used as an off-label medication for erectile dysfunction in urological settings (45). Therefore, as in patient B, trazodone may represent a valuable tool to be used as an antidepressant treatment in young people with depressive-anxious symptoms showing sexual dysfunctions when treated with SSRIs.

As described in Patient C, depression is a common comorbid diagnosis in PD patients (46). Often, differential diagnosis between characteristic PD symptoms and depressive symptoms may represent a hard challenge for clinicians, due to the overlap of specific manifestations, such as psychomotor slowing, fatigue, loss of energy and appetite, and difficulties in concentration (47). Moreover, anhedonia and cognitive symptoms, both specifically involving dopaminergic neurotransmission, seem to affect depressed PD patients more often as compared to non-comorbid ones (48, 49), thus challenging physicians to choose antidepressant treatments that may impact this specific cluster of symptoms, and, at the same time, trying to avoid possible interactions with the other drugs used to treat PD motor impairments. Although no proper clinical evidence exists, trazodone has been demonstrated to alleviate dyskinesia and behavioral alterations in animal models of PD (50), and 5HT2a/2c antagonism has been suggested to relieve depressive symptoms and motor impairment in PD patients (51). Finally, trazodone may be helpful in alleviating akathisia (52). Therefore, as in the last presented case report, trazodone may represent a valuable choice in depressed patients suffering from neuromotor diseases, whose depressive and motor symptoms (e.g. agitation, restlessness) may be exacerbated by pro-dopaminergic therapies.

## Conclusions

Depression may occur in the context of multiple comorbid pathologies, thus complicating diagnosis and treatment. The availability of a peculiar drug such as trazodone may help to tailor a personalized antidepressant treatment in poly-treated patients with several comorbidities.

This result may be achieved due to its malleability and a lack of interactions, as well the large number of formulations which permit multiple dosage adjustments and its use under particular conditions.

Although the present article might seem affected by a selection bias because of the apparent non-random selection of the cases, as previously described, they were specifically chosen as representatives of “real-world” patients with which clinicians usually deal in their daily practice. However, they were rigorously assessed, and the choice of treatment was based on broad clinical experience and fruitful debate amongst the selected experts in the field, in order to achieve the best consensus on treatment options.

More studies exploring the real-world conditions of antidepressants are desirable, with the aim of achieving the most personalized and tailored treatment of major depression in all contexts of its manifestation.

## Data Availability

The original contributions presented in the study are included in the article/**Supplementary Material**. Further inquiries can be directed to the corresponding author.
