# Supplementary material for: Case report: Personalizing the use of trazodone in real-world patients: a study of three cases of depression with comorbidities
Source: Front Psychiatry. 2024 Aug 29;15:1362221. doi: 10.3389/fpsyt.2024.1362221 (PMC11391422; doi:10.3389/fpsyt.2024.1362221)
Supplement: Supplementary file 1 [file Table1.docx]

**Table 1. NGT questions to experts for each round table**

**Case Report A**

**Question 1: Which kind of exam is indicated for a better characterization of the patient?**

| **Answer** | **RT I** | **RT II** | **RT III** |
| --- | --- | --- | --- |
| **a) Encephalic MRI** | **60%** | **0%** | **60%** |
| **b) Encephalic CT** | **10%** | **11%** | **40%** |
| **c) Blood exams** | **30%** | **89%** | **0%** |

**Question 2: What kind of therapeutic approach should be preferable in this case?**

| **Answer** | **RT I** | **RT II** | **RT III** |
| --- | --- | --- | --- |
| **a) Pharmacological treatment with tricyclic antidepressants** | **0%** | **0%** | **0%** |
| **b) Pharmacological treatment with non-benzodiazepine hypnotic** | **14%** | **0%** | **0%** |
| **c) Pharmacological treatment with multimodal antidepressants** | **86%** | **100%** | **100%** |
| **d) Psychotherapic and behavioral intervention** | **0%** | **0%** | **0%** |

**Case Report B**

**Question 1: How do you usually evaluate sexual dysfunction in your clinical practice?**

| **Answer** | **RT I** | **RT II** | **RT III** |
| --- | --- | --- | --- |
| **a) Open question during the interview** | **86%** | **100%** | **100%** |
| **b) Self-administered scales** | **14%** | **0%** | **0%** |
| **c) Hetero-administered scales** | **0%** | **0%** | **0%** |

**Question 2: What kind of antidepressant treatment switch would you make in case of sexual dysfunction?**

| **Answer** | **RT I** | **RT II** | **RT III** |
| --- | --- | --- | --- |
| **a) Switch amongst SSRIs** | **0%** | **10%** | **0%** |
| **b) SSRI 🡪 SNRI** | **0%** | **0%** | **0%** |
| **c) SSRI 🡪 TCA** | **0%** | **0%** | **0%** |
| **d) SSRI 🡪 SARI** | **100%** | **90%** | **100%** |

**Case Report C**

**Question 1: Considering patient's symptoms (tremor, bradykinesia, postural instability) which tests is indicated for greater diagnostic appropriateness?**

| **Answer** | **RT I** | **RT II** | **RT III** |
| --- | --- | --- | --- |
| **a) DAT-SCAN SPECT** | **100%** | **88%** | **100%** |
| **b) MRI** | **0%** | **12%** | **0%** |
| **c) EEG** | **0%** | **0%** | **0%** |

**Question 2: In the presence of some persistent symptoms in the patient receiving trazodone OAD 150 mg/day, which therapeutic options would you choose from those indicated?**

| **Answer** | **RT I** | **RT II** | **RT III** |
| --- | --- | --- | --- |
| **a) Switch to another antidepressant drug** | **0%** | **0%** | **0%** |
| **b) Adding a benzodiazepine** | **0%** | **0%** | **0%** |
| **c) Raising dosage of trazodone to 300 mg/day** | **86%** | **88%** | **100%** |
| **d) Other** | **14%** | **12%** | **0%** |

**RT=round table; MRI=magnetic resonance imaging; CT=computed tomography; EEG=electroencephalogram; SSRI=selective serotonin reuptake inhibitor; SNRI=selective noradrenalin reuptake inhibitor; TCA=tricyclic antidepressant; SARI=** **serotonin antagonist/reuptake inhibitor; SPECT=** **Single-photon emission computed tomography**
